# Integrating stakeholder feedback into the design of a peer-delivered primary care wellness program: A rapid qualitative study

**DOI:** 10.1186/s12913-023-10324-x

**Published:** 2023-12-07

**Authors:** Emily M. Johnson, Kyle Possemato, Matthew Chinman, Gala True, Jacob Hedges, Brittany N. Hampton, E. Jennifer Edelman, Stephen A. Maisto

**Affiliations:** 1 VA Center for Integrated Healthcare, Syracuse VA Medical Center, 800 Irving Ave. (116C), Syracuse, NY 13210 USA; 2 Center for Health Equity Research and Promotion, VA Pittsburgh Healthcare System, Pittsburgh, PA USA; 3 RAND Corporation, Pittsburgh, PA USA; 4 VISN 4 Mental Illness Research, Education, and Clinical Center, Pittsburgh, PA USA; 5 South Central Mental Illness Research Education and Clinical Center, Southeast Louisiana Veterans Health Care System, New Orleans, LA USA; 6 Department of Medicine, School of Medicine, Louisiana State University Health Sciences Center, New Orleans, LA USA; 7 Department of Internal Medicine, Yale School of Medicine and Department of Social and Behavioral Sciences, Yale School of Public Health, New Haven, CT USA; 8 Department of Psychology, Syracuse University, Syracuse, NY USA

**Keywords:** Patient-centered care, Primary health care, Delivery of health care, Veterans, Qualitative research

## Abstract

**Background:**

Individuals seen in Primary Care with behavioral health concerns who decline behavioral health treatment may benefit from the support of peers (consumers in recovery from behavioral health concerns employed to support other consumers). *Whole Health STEPS* is a new intervention for Veterans in Primary Care with behavioral health concerns which combines essential elements of peers’ role and the Whole Health model using a stepped-care design. We incorporated stakeholder feedback in the *Whole Health STEPS* design to improve fit with Veterans, peers, and primary care settings.

**Methods:**

We conducted semi-structured qualitative interviews with VA staff using questions derived from the Consolidated Framework for Implementation Research (CFIR). Participants were recruited via a maximum variation strategy across a national sample and interviewed between January 2021-April 2021. The analytic design was a rapid qualitative analysis. Interviews addressed design decisions and potential barriers and facilitators to future implementation. Then, we made adaptations to *Whole Health STEPS* and catalogued changes using the Framework for Adaptations and Modifications-Enhanced (FRAME). A VA peer conducted the interviews, participated in analyses, assisted with design modifications, and co-wrote this paper.

**Results:**

Sixteen staff members from 9 VA primary care peer programs participated (8 peers and 8 supervisors/administrators). Feedback themes included: capitalizing on peer skills (e.g., navigation), ensuring patient-centered and flexible design, and making it easy and efficient (e.g., reducing session length). Understanding the structure of primary care peers’ roles and their interactions with other programs helped us identify role conflicts (e.g., overlap with Whole Health Coaches and Health Behavior Coordinators), which led to design modifications to carve out a unique role for *Whole Health STEPS*. Staff also made recommendations about marketing materials and training tools to support *Whole Health STEPS* roll out.

**Conclusions:**

Feedback from frontline staff, including peers, in the design process was crucial to identifying essential modifications that would not have been possible after initial trials without re-evaluating efficacy due to the extent of the changes. *Whole Health STEPS* was adapted to fit within a range of program structures, emphasize peers’ unique contributions, and streamline delivery. Lessons learned can be applied to other interventions.

**Supplementary Information:**

The online version contains supplementary material available at 10.1186/s12913-023-10324-x.

## Introduction

Patients seen in primary care with untreated behavioral health concerns such as depression have worse behavioral health outcomes including poorer treatment response, remission rates, and chronicity [1]. Individuals with behavioral health concerns also have high rates of co-morbid physical health concerns that often go untreated [2]. In Integrated Primary Care settings, routine clinical screenings for behavioral health concerns identify individuals with unmet needs and prompt clinicians to offer referrals or warm handoffs [3]. This recommended workflow is well-suited to identify and meet the needs of individuals with mental health concerns [3]. However, even in well-functioning systems, patients decline or discontinue care. For example, following a Department of Veterans Affairs (VA) primary care screening for posttraumatic stress disorder (PTSD), 39% of Veterans who screened positive did not receive a referral or prescription for behavioral health services, and at least 19% of those individuals declined a referral [4]. Offering non-symptom and non-diagnostic focused services in alternate formats may be a strategy to engage patients who otherwise decline behavioral health services to support their overall wellness.

Patients seen in primary care who have behavioral health symptoms and decline behavioral health services may benefit from services delivered by peer specialists. Peer specialists, also known as peers, are typically defined as consumers in recovery from mental health or substance use concerns who have training and a formalized role to help others in recovery [5, 6]. Key ingredients of peer services include “1) social support, 2) experiential knowledge, 3) trust, 4) confidentiality and 5) easy access” [6] (p2). The evidence base for the value of peer services in mental health settings suggests potential benefits in patient experience (e.g., empowerment, patient activation), mental health symptoms, and healthcare utilization (e.g., better relationship with providers, better engagement with care) [5, 7, 8]. Increasingly, peer services are expanding beyond mental health clinics into new settings like Primary Care [9–12]. Also, peers are expanding their roles into spheres like wellness coaching [9–12]. As peers move into new settings and roles, it is important to evaluate these innovations to ensure they maintain essential elements of peers’ profession.

Peer-delivered health and lifestyle interventions for individuals with mental health concerns (e.g., *In Shape*, *Living Well*, Peer Delivered *Whole Health Coaching*) demonstrate promising results, but warrant additional research [13–17]. Many of these interventions (e.g., *In Shape* and *Living Well*) [13–15, 17–19] were developed for specialty mental health settings and are lengthy (e.g., 8–24 sessions 40–75 min each), which may not fit well within Primary Care, or involve groups, which has been found to be a barrier to treatment [20]. To our knowledge, no individual peer-delivered wellness coaching interventions have been specifically tailored for Primary Care settings for patients with behavioral health concerns who are not engaged in behavioral healthcare. A study evaluating peer-delivered Whole Health Coaching for Veterans in a VA primary care clinic with PTSD suggested variability in participant response: some participants started to improve with assessment only, some participants improved with the intervention, and some participants failed to respond [16]. This variability suggests that monitoring response and tailoring the intensity of support from low to high intensity with options for referral to different services as needed may be one option to increase efficiency and improve fit within primary care.

Stepped-care is a healthcare delivery model in which response to treatment is assessed at pre-specified timepoints to determine whether to step-up or step-down the intensity of services [21]. The recommended treatment approach is to provide the lowest intensity treatment likely to provide meaningful improvement and adjusts as needed [21]. Stepped-care interventions have demonstrated efficacy for a range of concerns including depression and alcohol use [22–25]. However, the literature suggests that putting stepped-care interventions into practice can be challenging and can benefit from intentional implementation support [26–28]. Therefore, it is crucial to consider future implementation and dissemination plans in the design of new stepped-care interventions to maximize likelihood of success.

We developed a peer-delivered, patient-centered, stepped-care wellness intervention for Veterans in primary care with mental health concerns. We developed the intervention using Design for Dissemination, a concept in implementation science that can increase the likelihood of future uptake, implementation success, and sustainment of new initiatives [29]. A key principle in the Designing for Dissemination process is involving stakeholders as early as possible utilizing implementation science frameworks [29, 30]; we involved a Veteran peer in all parts of the research process to ensure peer perspectives were reflected throughout. Designing for Dissemination can, in part, address the well-known gap between research and implementation [31] by identifying and addressing potential implementation problems up front in the design phase. Thus, the overarching goals of this paper are to present what we learned from 1) a rapid qualitative study to better understand the needs of key staff stakeholders and 2) the systematic process of integrating staff feedback into the design of a new peer service.

## Methods

In keeping with the goal of promptly and directly incorporating stakeholder input into the intervention design, we used a rapid qualitative approach, a qualitative design which often involves semi-structured data collection around defined themes, a data reduction approach to analysis without the in-depth coding of many other qualitative methods, and is tailored to allow timely integration of results in an active study or healthcare setting [32]. Between January 2021 and April 2021, we conducted semi-structured qualitative interviews with peers and supervisors/administrators from VA primary care peer programs and analyzed the data using an iterative, team-based approach. A Veteran who previously worked as a VA peer (JH)^1^ was a core research team member involved in data collection, analysis, interpretation, design decisions, and authorship. Having a peer on the research team is consistent with the Peer Specialist Research Agenda and VA recommended guidelines for Veteran involvement in research [33, 34]. We followed reporting guidelines for the American Psychological Association Journal Article Reporting Standards (JARS) for qualitative designs [35]. This study was determined by the Syracuse VA IRB to be exempt and approved by the Syracuse VA Research and Development committee as an exempt project.

### Initial *Whole Health STEPS* design

*Whole Health STEPS* is a new intervention using a peer-delivered, patient-centered, stepped-care, wellness approach to support Veterans in primary care with behavioral health concerns who are not engaged in behavioral health treatment. We integrated existing programs and elements, including the pre-defined peer role and Whole Health model into a comprehensive package for delivery. Although no final decisions had been made about the design’s details, we approached interviews with initial design ideas to prompt feedback. The concept was based on work from a peer-delivered wellness coaching pilot trial, which found that peer-delivered wellness coaching had high patient satisfaction and helped participants make progress on individualized wellness goals [16]. One observation was that regular assessment of wellness goals (a component of the research design) benefitted some participants with a much lower intensity service (assessment only) than peer-delivered coaching (the intended intervention) [16]. Hence, our idea was to combine the assessment and coaching service into a stepped-care design which allows individual dose tailoring to minimize patient burden and streamline service delivery.

Essential elements for this intervention included peer-delivery, primary care setting, Whole Health focus, stepped-care design, use of a semi-structured interview-based tool to guide decision-making, and target population of Veterans with behavioral health concerns who were unengaged in behavioral health services. As core elements, we believed they should remain, but were open to revising them to optimize implementation (e.g., adjusting language, re-packaging). Whole Health is the VA’s model for delivery of patient-centered healthcare that focuses on overall health and well-being [36]. The intervention name, *Whole Health STEPS* (Structured Tiered Engagement with Peer Support) was pre-determined based on these elements. We described additional thoughts about the possible structure (e.g., proposed components, sample questions for the structured interview tool) to participants (see Supplement for participant handout). An initial manual draft was used as a starting place for revision.

### Participants

A maximum variation sampling approach [37] was used to identify participants working in VA primary care peer programs. Sites were identified through the national primary care peer program to recruit sites with variation in key characteristics likely to influence future implementation of *Whole Health STEPS*, including the nature of the primary care peer program (e.g., types of services provided and administrative structure), geographical region, Veteran population served (e.g., rural/urban), type of medical facility (e.g., major medical center, community outpatient clinic), and related programs (e.g., Primary Care, Whole Health, Primary Care Mental Health Integration, etc.). Participants were recruited through an email introduction and screener for interested parties to ensure eligibility (either a peer working in a VA primary care setting or a supervisor/ administrator of a VA primary care peer program). Although the participants may have incidentally had a relationship with the researchers at the time of recruitment, no intentional relationship was established prior to the interviews.

A priori estimates suggested 12–24 participants (6–12 from each group [peer/ supervisor or administrator]) would be sufficient for saturation given the research question scope and sample homogeneity [38]. The final sample size was determined through research team consensus that data saturation (whether new data are repeating old data) and a priori thematic saturation (whether identified themes are exemplified in the data) were sufficient to capture the most salient concepts [39, 40]. Data saturation and a priori thematic saturation were systematically evaluated through prompts on the rapid qualitative summary template used in the iterative analysis process. Saturation was also evaluated as a team during the iterative matrix analysis in which summaries from the first round of recruitment were compared with summaries from the second round of recruitment.

### Data collection

#### Semi-structured VA staff stakeholder feedback interviews

**Fig. 1:**
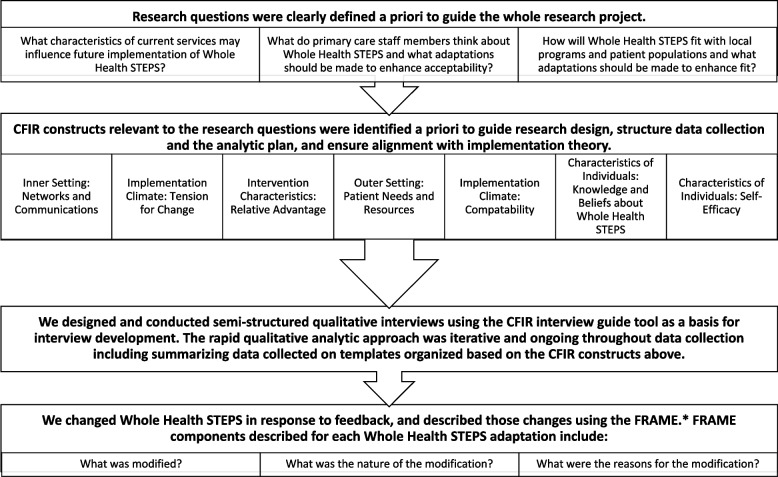
Methods flow chart. Note. Elements of FRAME consistent across all adaptations which are not described individually but rather throughout the methods include: “When did the modification occur?”, “Were adaptations planned?”, “Who participated in the decision to modify?”, “At what level of delivery?”, and “Relationship fidelity/core elements?”

Data were collected in individual semi-structured interviews (24–42 min long). We identified relevant implementation constructs from the Consolidated Framework for Implementation Research (CFIR) based on our research questions; CFIR was developed as a practical guide for systematically identifying and assessing barriers and facilitators to new program implementation (Fig. 1) [41, 42]. Additional questions were added to get direct feedback about specific aspects of *Whole Health STEPS* design, including the stepped care decision points and delivery format. Interviews were conducted via telephone and audio recorded. Interviews were conducted by the Veteran research staff member (an experienced qualitative interviewer) and observed by a research assistant who took detailed notes, transcribing as much of the interview as possible in lieu of making a verbatim transcript. Templated summaries were completed directly from audio recordings and field notes [43]. Participants were not re-contacted after the interview was concluded. The interview was pilot tested with research staff prior to data collection. The template did not include systematic self-disclosure from the interviewer although information may have been conveyed incidentally through the course of conversation.

#### Tracking Adaptations

Changes made to *Whole Health STEPS* in response to stakeholder interviews, feedback from the Veteran research staff member, and other sources were tracked by saving iterative versions of the manual and maintaining notes about decisions. Adaptations were listed in a table structured based on concepts from using the Framework for Reporting Adaptations and Modifications-Enhanced (FRAME) (Fig. 1) [44].

### Data analysis

#### Qualitative analysis of VA staff stakeholder feedback interviews

Data analysis used rapid qualitative analysis based in a matrix analysis approach followed by the Sort and Sift, Think and Shift approach [32, 45, 46]. Analysis was iterative and ongoing throughout data collection to assess saturation and allow early identification of key information (e.g., recommendations to make adaptations to key elements of *Whole Health STEPS*). Rapid qualitative analysis uses a data reduction approach which uses a template to summarize responses for each interview within pre-specified domains corresponding directly to interview questions, then analyzes all the summaries as a group using the matrix analysis approach. Domains included each of the 7 CFIR domains (Fig. 1), direct feedback about *Whole Health STEPS*, and space for other notes. Per CFIR coding guidance, the summary template also included instructions to rate strength (the degree to which implementation was facilitated or hindered, with possible choices being 0, 1, or 2) and valence (positive or negative impact on implementation) of participant responses [41]. These ratings assisted with interpretation of the relative emphasis of each comment in the summary when considering each statement in the context of the remainder of the interview and summaries from other participants. Questions were added at the end to help staff consider whether saturation had been achieved. Analysis was conducted in Microsoft Access.

Three staff members acted as the primary analytic team, the PI, the Veteran staff member who also conducted the interviews, and the research assistant who did note-taking during the interviews. Each of these staff members practiced completing summaries using the summary template on two role-plays to establish consistency and hone the instructions. They then summarized the first interview along with two more interviews identified as challenging to summarize. The PI provided feedback to ensure consistency and accuracy in CFIR domain definitions. A consensus process was used to resolve discrepancies and establish consistency across the raters in writing objective, comprehensive, and brief summaries of interview content.

Summaries were routinely viewed in matrix format, allowing comparison and synthesis across interviews within each domain [45]. Regular review of the matrix enabled discussion of gaps in information, saturation, variability within domains across interviews, emerging themes, actionable feedback, and researcher inferences from the data. The PI developed an overall summary reflecting content within each domain for team review. After the team reached consensus within each domain, each member of the analytic team independently generated a short list of major themes across domains without reviewing the data, recalled responses which changed how the team thought about the *Whole Health STEPS* design, noting any new major themes, and then re-reviewed the summaries to take note of high impact statements, noting whether they fell within previously identified themes or reflected new themes. The team discussed independently generated themes and reached consensus on the final cross-cutting themes. A visual diagram of key results was collaboratively generated and shared with the remainder of the authorship team (including experts in qualitative methods, peer services, integrated primary care, and Veteran engagement) for feedback and verification.

#### Analysis of adaptations

Adaptations were analyzed using narrative description of text-based data (i.e., descriptions and actions taken) and counts/frequencies of categorical data (i.e., what was modified, type/nature of adaptation, and reasons for adaptation). Categories were based on the FRAME with minor changes to reflect the data. The PI independently described and categorized each adaptation. Summaries and categories were subsequently reviewed and confirmed by the full authorship team.

## Results

We contacted staff at 14 programs (*n* = 56 staff). Seventeen participants completed email screeners (100% eligible). Sixteen staff members participated in interviews (29% response rate; *n* = 16, 8 peers and 8 supervisors/administrators from 9 VA peer in primary care programs). Participating sites were recruited from a range of geographic regions of the US, types of medical facilities (e.g., large and small, with and without specialty medical services), and rurality (serving 5.4%-69.3% rural areas). Approximately half the participants self-identified as female and approximately half the participants self-identified as White/Caucasian. Other racial and ethnic identities represented included Black, Hispanic, and mixed race.

### Stakeholder feedback about design and implementation considerations

Participant responses about implementation of their programs, local site characteristics, and feedback about *Whole Health STEPS* were summarized by a priori CFIR construct to illustrate essential concepts (Table 1).

**Table 1:** Summary of stakeholder feedback within each CFIR construct assessed

**CFIR Constructs** (abbreviated definitions)**/** **Stakeholder Feedback Summary**	**Exemplars**
**Inner Setting: Networks and Communications** *(the nature and quality of social networks and communication in an organization)* Overall, both peers and supervisors reported strong working relationships. Some communication challenges included: • Other team members lacked understanding of peers’ role • Generating sufficient referrals • Staying connected when peers cover very large teams or numerous teams or are doing remote work • Finding time for conversation with busy providers Effective communication strategies included: • Peers marketing their services to providers and providing education • Building individual working relationships through being present virtually and in person • Attending team meetings • Peers demonstrating value and reducing provider burden through assisting with cases • Peers direct marketing to Veterans by spending time in waiting rooms and approaching Veterans • Open, flexible, multimodal communication (e.g., in person, MS Teams, phone) • Having established team members as champions	“At the very beginning, nobody knew who I was or what I could do, nobody was really interested, now to build those relationships, I just pretty much just showed the value of peer support.” (Peer) “Communication wise it can be a little bit unclear sometimes, what their role is, what they should do, what they shouldn’t do. (Supervisor/Administrator)
**Implementation Climate: Tension for Change** *(stakeholders’ perceptions of whether their current situation needs to change)* There was high variability in tension for change. Necessary changes/concerns with the current situation included: • Role confusion (e.g., between peers and Whole Health Coaches, peers and Primary Care Mental Health Integration providers, etc.) • Duplication in programming and confusion about appropriate referrals to/use of programs • Separation between programs both in terms of staff perceptions (e.g., considering tasks another person’s job) and administrative organization (e.g., separate staffing, different position descriptions for similar levels of staffing, different billing requirements) • Incomplete implementation of programs (e.g., not having all program elements available) Reasons to maintain the status quo rather than adopt new programming: • Competing priorities and time constraints for both providers and Veterans • Resistance to change among staff Strategies to address concerns driving desire to change the current situation: • Communication, marketing, and training for staff about programs including individual feedback about referrals and concrete guidance • Increasing peer presence to increase familiarity • Coordination between national and local staff about program scopes and how to integrate/coordinate programs • Ensure adequate provider-level and supervisory staffing for programs	“Integrating Whole Health into all levels of care has had some challenges. There’s some inherent rub, specifically, with HPDP and what their healthy living messages are and their modality of how they treat folks and then WH comes in with somewhat similar but somewhat different kind of ways of approaching that and how they integrate. So there’s some conflicting missions and goals within the programs we have here… I think there’s some turf wars around that, whose job is it, and what they are doing so I think Whole Health has struggled a little bit to integrate into different programs.” (Supervisor/Administrator) “I think people will tell you yes that whole health is great. But the other thing folks are going to tell you is they are incredibly overworked and understaffed. They are just trying to stay afloat. Anything new-one more thing- even minute little thing is incredibly overwhelming-Anything we can do to support staff but not create more work for them. That is the greatest barrier.” (Supervisor/Administrator)
**Intervention Characteristics: Relative Advantage** *(comparative advantage of WH-STEPS vs. the status quo)* Overall, participants perceived Whole Health STEPS as similar in value to existing programs, but some staff perceived Whole Health STEPS as better or worse. Actual and potential disadvantages to Whole Health STEPS included: • Some of the steps and step-transitions were perceived as more complicated and less efficient • Potential inconsistencies with how peers perceive their role, what they prefer to do, and strengths of peers as providers (e.g., recovery model) • Duplication of services and role confusion could occur with Whole Health Coaching • Might reduce peer availability to support mental health needs vs. Whole Health needs • Rigid structure Actual and potential advantages to Whole Health STEPS included: • Brief telephone contact as a level (step) of care • Having peers work at the top of their scope and take tasks currently being performed by licensed independent providers • Having a peer provider can increase Veteran buy-in • Patient-determined goals • The structure is beneficial to monitor outcomes and increase comfort for Veterans and peers	“Just based off my normal interactions with Veterans, it’s similar, but having it formalized, regimented, makes it easier to dictate the outcomes a little bit better just because there’s levels and checkpoints that fall into the STEPS program that would be beneficial.” (Peer) “I think what we miss is some of the other work the peers are doing right now. We are missing review of the recovery model with our patients [and] … I like that it’s structured but I wish that it offered more flexibility for patients to build rapport with their peers as well, I think that’s really important in the first session.” (Supervisor/Administrator)
**Outer Setting: Patient Needs & Resources** *(how WH-STEPS meets the needs of Veterans and barriers/facilitators to Veterans participating in WH-STEPS)* Overall, Whole Health STEPS was perceived as a good fit to Veterans’ needs: • Structure enabling Veterans to know expectations • Tool will ensure more comprehensive assessment • Veterans have increased trust with peers compared to other staff • Encourage setting and following-up on self-identified goals • More support than comparable interventions • Making basic changes to lifestyle may be sufficient to address some Veterans’ needs without referral to a higher level of care or prepare them for a high level, if needed • Addresses loneliness/isolation • Virtual care options increase flexibility in scheduling Potential barriers to Veteran engagement were noted: • Generating referrals and increasing visibility • Hour long sessions • This will be a difficult population to engage because supporting Veterans with low motivation for change and making health and lifestyle changes are challenging • Access for Veterans who are not available during normal working hours • Some Veteran populations may have difficulty engaging due to more immediate needs (e.g., homelessness, difficulty establishing basic healthcare) • Lack of access to a working phone is a barrier to telephone-based services for some Veterans • Some Veterans will not want virtual care options	“I just wonder about advertisement and how we can make this more accessible/visible so that we are getting the good turnout. This is like preventative medicine stuff. I think anyone can benefit from this.”(Supervisor/Administrator) “I do think some patients aren’t sure what to talk about or how to use a mental health provider or a peer and I like that it can provide a framework for what their sessions could look like.” (Supervisor/Administrator)
**Implementation Climate: Compatibility** *(fit with stakeholders’ values, needs, and workflow)* Overall, Whole Health STEPS was perceived as consistent with participants’ values and models of care: Structure was perceived as helpful to complement other organizational changes (e.g., transformation to high reliability organization) • Good fit with goals and content of existing programs including PC, PCMHI, and Whole Health Concerns related to compatibility included: • Less efficient than existing processes • Would result in duplication of services and role confusion/conflict due to administrative separation between Whole Health Coaching and Peer programs and positions • Insufficient referrals to support the service • Potential for Whole Health STEPS to take away from other peer functions (e.g., connecting Veterans to care)	“It would allow me to still do everything else I’m doing. This would just be another sort of interaction, another tool that I would be able to interact with a particular vet, rather than an add on or another box to check, or another ball to juggle. It’s just another tool that I’d get to utilize.” (Peer) “Blending peer specialists doing the whole health steps in their two different departments…we strive to have a great working relationship but it might confuse the role.” (Supervisor/Administrator)
**Characteristics of Individuals: Knowledge and Beliefs about Whole Health STEPS** *(general attitudes about Whole Health STEPS)* Participants generally expressed positive beliefs and attitudes about Whole Health STEPS although enthusiasm varied. Specific attributes which contributed to their perceptions largely reflecting concerns (e.g., less efficient) and strengths (e.g., structure, peer focus, flexibility, brief telephone appointments) noted above	“Honestly, I think it’s just stressful…” (Peer) “All the steps I love, I think that’s great and it certainly plays on the strengths that peer support can bring.” (Peer)
**Characteristics of Individuals: Self-Efficacy** *(belief in own capabilities and training needs to achieve confidence)* Both supervisors and peers generally believed they could be effective at implementing Whole Health STEPS. They recommended some specific training needs and training preferences including using role-plays, case examples, on-going consultation. Respondents also wanted a detailed manual describing the steps and providing tips and guidance on delivery, especially tailoring, personalizing, and building/maintaining rapport. A listing of available services was also requested	“I would feel perfectly confident. This is how I base my whole interaction with them [Veterans].” (Peer) “One thing I would love to see is more examples. My 2 guys [peers] are very concrete. If it’s left in ambiguity, I tend to lose them a little. It’s just their own learning style, which we all learn a little bit differently and that’s just fine.” (Supervisor/Administrator)
**Other Feedback about Whole Health STEPS and Implementation** Several participants noted the current format doesn’t fully capitalize on peer qualities or explain why peers are essential to delivering Whole Health STEPS (e.g., peer credibility, stigma-busting) which may impact peer satisfaction, Veteran experience, and contribute to role confusion Participants provided feedback on how to approach the decision-making process for step-changes for Whole Health STEPS and described current practices for peers making level-of-care decisions with Veterans including: • Decision criteria need to be clear and concrete but allow for flexibility for individual Veteran needs and peer judgement • Referral is an important element but it should not be a part of the stepped care process; it is a core peer service that should be immediate • Both objective indicators (e.g., the Whole Health goal assessment) and subjective experience (e.g., not making progress) are useful to inform step and navigation decisions Participants also provided feedback and thoughts about important considerations in delivery of Whole Health STEPS including: • Telehealth formats including telephone and video telehealth will need to follow established guidelines and require specific training • Veteran handouts with Whole Health STEPS information would be helpful • An intermediate step between 15-min telephone sessions and hour-long sessions was recommended • Tracking caseload and managing multiple contacts was identified as a potential challenge particularly for a stepped-care approach with Veterans at different steps and stages of care • Some participants wanted to integrate groups into Whole Health STEPS • Peers may have low satisfaction with the Whole Health goal assessment if it feels like “grunt work” • Peers need to be aware of safety and other issues outside of their scope and if self-management is insufficient	“Peer specialists are very unusual employees and the advantage that they bring is tied directly to their personal experience, the credibility they have, and the knowledge they have as someone who has personally navigated the system. When I look through this, I don’t see any reason why this is tied to a peer.” (Supervisor/Administrator) “I think in general, yes, we should have someone else involved in that [step decisions] because you know peers don’t refer.” (Peer) “I was a medic in the military, so you never discontinue care until someone else is there to be able to continue. We still track their care so once they’re done, we’re still part of a team and then once they are stabilized they come back to us for peer support services.” (Supervisor/Administrator)

Bold underlined text reflects the constructs assessed largely drawn from the Consolidated Framework for Implementation Research (CFIR). *Italicized text* reflects the abbreviated definition from CFIR

Participant responses highlighted three cross-cutting themes across CFIR constructs: 1) the importance of accentuating peers’ role and unique strengths, 2) the need to emphasize patient-centered aspects of the Whole Health model, and 3) the need to make it easy and efficient for primary care peer delivery. Additionally, interviews raised a design flaw, specifically, 4) role conflicts in staffing and administrative program structure made the original design incompatible with some VA sites.

#### Cross-cutting theme: the importance of highlighting peers’ role and unique strengths

Participants expressed that the original design did not “capitalize” enough on the peers’ role. They noted important elements that differentiate a peer from other clinical providers, such as “being able to be an example of recovery” and “helping connect Veterans” should be included in the intervention. To keep the intervention true to the peer specialist discipline and emphasize why peers should deliver this intervention, participants suggested making peers’ role more central by highlighting their “Veteran identity,” “shared experience,” and “credibility.” Participants described how this modification would increase not only peers’ job satisfaction and willingness to deliver *Whole Health STEPS*, but also Veterans’ willingness to participate. One participant noted that having “something that the peer specialists can own and do” would increase buy-in from Veterans, specifically from “folks that aren’t established in mental health.” To find balance between the integrity of the intervention and incorporation of the peers’ role, one participant suggested giving peers flexibility with the intervention “so they can shine and do what they are trained to do.” Other participants suggested providing clear guidance for peers in formats geared towards their learning styles, such as “case examples,” “vignettes,” and “role-plays.”

#### Cross-cutting theme: need to emphasize patient-centered aspects of the whole health model

The patient-centered nature of the intervention received support; participants voiced that this aspect should be emphasized in the program moving forward. Respondents highlighted the importance of using feedback from Veterans to inform decisions about the level of care and referrals to ensure the Veterans’ needs were being met. Additionally, one participant stated “the ultimate goal is to step down” in care once Veterans reach goals. Another participant commented that the emphasis on self-identified goals, a key component of patient-centered care, would improve Veteran engagement, especially for those not engaged in mental health. “…providing them the services they might need and again on their self-identified goals… which is something beautiful that whole health does, and you’ll get a lot of buy-in with that.”

#### Cross-cutting theme: make it easy and efficient for primary care peer delivery

Participants shared the importance of making *Whole Health STEPS* easy, efficient, and compatible with primary care peer services. 60-min sessions were perceived as “long and drawn out” and would “eat up precious time” compared to existing practices. Participants suggested that 30 min was an appropriate length for similar health coaching and peer sessions, and 30-min orientation sessions were a perceived benefit of the proposed program structure. A participant also noted the importance of the 15-min brief calls to reduce burden on peers and Veterans, stating the brief calls would be “minimal.”

The structure of *Whole Health STEPS* was positively received and highlighted as unique. One participant noted how the “regimented” structure allowed Veterans to “know what’s coming next.” However, participants noted the importance of keeping the program simple to fit within peers’ workflow. One participant also suggested that to keep peers from feeling “a little lost” when managing their caseload and monitoring Veteran progress, a tracking system would be helpful.

#### Design flaw: roles need to be better aligned with structure of peer programs

Participants noted concerns with implementing *Whole Health STEPS* given the administrative structure of some existing programs: “I don’t think this is a good fit for our site. Our programs are separate.” Examples of separate programs with similar functions across different sites included the Whole Health program and Health Promotion and Disease Prevention program. Participants explained that the way the original design was structured, peers would be taking on responsibilities specifically assigned to other staff members from other programs at some sites (e.g., Whole Health Coaches and Health Behavior Coordinators both have defined health coaching roles) that would “cause more confusion” and “duplication of services.” Different sites noted different implementation barriers related to how programs and roles were structured but one example was that sites that employed both peers and Whole Health Coaches in primary care had concerns about peers providing the same service as the Coaches. The barriers inherent to the original design were described with a range of severity, with one participant noting that the conflicts could be “navigated pretty well if there’s goodwill,” but other participants explained that the original design would completely prevent implementation at their site. Suggestions included not having peers conduct formal coaching and focusing on the peer role. “I’d go back to that issue of their specific role.”

### Adaptations to *Whole Health STEPS* in response to stakeholder feedback

Discrete changes made between the initial and revised manual were summarized (*n* = 27) and classified using the FRAME (Table 2). Changes were predominantly made to the content (*n* = 10) and training and evaluation materials (*n* = 10), with a few changes made to implementation and scale-up activities (*n* = 5) and format (*n* = 2). The types of changes made were mostly tailoring, tweaking, and/or refining materials (*n* = 16), but a few changes were made to adjust packaging or materials (*n* = 5), add elements (*n* = 3), loosen structure (*n* = 2), remove elements (*n* = 1), and substitute elements (*n* = 1). The goals of these changes were primarily to increase the likelihood of future implementation success. Changes were in response to a wide range of contextual factors spanning organizational considerations at the VA or clinic level (*n* = 12), provider considerations for peers as providers (*n* = 16), and recipient considerations for a Veteran population (*n* = 9).

**Table 2:** List of all adaptations made to Whole Health STEPS with Framework for Adaptations and Modifications-Expanded (FRAME) classifications

What is modified?	Adaptation Number & Description	Details and Actions Taken	Type/Nature of Adaptation	Goal & Contextual Reasons for Adaptation
Content	Provide concrete guidance for peers on how to apply the Whole Health model	Operational definitions of Whole Health skills and recommendations on how to apply them in the context of Whole Health STEPS were added to the manual	Tailoring / tweaking / refining	*Goal:* Improve fidelity *Contextual Reasons:* Peer training and skills
	Remove elements which duplicate existing programming (e.g., Whole Health Coaching)	“Whole Health Coaching” sessions were replaced with “Peer Support for Whole Health” which involves supporting Whole Health in a primary care setting within a peer scope of work. The substitution has equivalent intensity but uses a peer model rather than a coaching model which has been rolled out in other programs with other staff	Substituting	*Goal:* Improve adoption *Contextual Reasons:* VA/clinic competing demands or mandates
	Incorporate patient navigation as a central element	Remove referral as a step or level of care and instead incorporate patient navigation, as a core element to be used at all levels for all Veterans	Removing/ skipping elements & Adding elements	*Goal:* Improve effectiveness/ outcomes *Contextual Reasons:* Peer cultural norms/ competency & Veteran access to resources
	Increase emphasis on peer role and skills (e.g., recovery model)	The manual was updated to operationally define peer skills and indicate how to apply them in Whole Health STEPS	Tailoring/ tweaking/ refining	*Goal:* Improve adoption *Contextual Reasons:* Peer cultural norms/ competency
	Add explicit focus on relationship building including strategies to build rapport with structured interventions	Sections on relationship building and how to build/maintain rapport with brief, time-limited, structured, interventions were added to the manual	Tailoring/ tweaking/ refining	*Goals:* Increase satisfaction & Increase retention *Contextual Reasons:* Peer cultural norms/ competency
	Decrease structure & Maintain structure	Because structure was perceived as both positive and negative for peers and Veterans, manual revisions focused on ensuring a semi-structured approach. Instructions were added on how to personalize Whole Health STEPS to peer and Veteran preferences	Tailoring/ tweaking/ refining	*Goal:* Increase satisfaction *Contextual Reasons:* Peer perception of intervention & Veteran preferences
	Ensure the Whole Health goal assessment tool fully supports delivery of peer services	The assessment tool was revised to ensure that all elements peers need to monitor are present (e.g., prompts for safety concerns, social needs, etc.)	Tailoring/ tweaking/ refining	*Goal:* Improve fit with VA/ clinic setting *Contextual Reasons:* VA/ clinic regulatory compliance
	Include Veterans in step decisions along with indicators of subjective and objective progress	Step decision criteria were revised to explicitly include Veterans’ perspectives on step decisions, objective data from the Whole Health goal assessment tool, and peer autonomy to include subjective perceptions of progress	Tailoring/ tweaking/ refining	*Goal:* Improve fit with recipients *Contextual Reasons:* Peer clinical judgment & Veteran motivation and readiness
	There need to be decision criteria about decreasing and discontinuing support to increase autonomy and ensure time-limited episodes of care	The decision criteria were revised to include guidance on when and how to taper and discontinue Whole Health STEPS	Adding element	*Goal:* Improve feasibility *Contextual Reasons:* VA/ clinic service structure
	Include Veteran handouts to provide information about Whole Health STEPS	A Veteran handout with an overview of the Whole Health STEPS program and outline of what to expect during the Whole Health goal assessment was developed	Changes in packaging or materials	*Goal:* Improve fit with recipients *Contextual Reasons:* Peer preferences & Veteran ease of access/ utilization
Contextual (format)	Increase flexibility in scheduling options (e.g., modality, frequency, and length) to better meet Veterans’ needs and reduce barriers	Scheduling flexibility was increased with recommended ranges rather than specified frequencies and session lengths. Modality recommendations will be continually updated to keep recommendations up to date with current regulatory, legal, and best practice guidelines	Loosening structure	*Goal:* Improve fit with recipients *Contextual Reasons:* Peer preferences & Veteran ease of access/ utilization
	Reduce the length of the hour-long sessions	The recommended session length for full length peer sessions is now 30–60 min	Loosening structure	*Goal:* Improve feasibility *Contextual Reasons:* VA/clinic service structure, Peer preferences, & Veteran ease of access/ utilization
Implementation and scale-up activities	Develop sample scripts for peers, brochures, and other marketing materials to explain Whole Health STEPS to Veterans and assist with referrals and educating providers	Marketing scripts and materials for the research trial will be translated to clinical application during implementation	Changes in packaging or materials	*Goal:* Increase reach/ engagement *Contextual Reasons:* VA/clinic social context & Veteran motivation/ readiness
	Clearly define appropriate referrals for Whole Health STEPS and ensure easy referral processes for Veterans and providers	Marketing materials describing the intended population and providing clear, simple instructions on how to refer/enroll developed for research will be translated to clinical application during implementation	Changes in packaging or materials	*Goal:* Increase reach or engagement *Contextual Reasons:* VA/clinic service structure & Veteran ease of access/ utilization
	Incorporate in person training and consultation with successful sites into the training plan	The competency-based training plan referenced above includes hands-on in-person training activities. Recommendations for consultation were noted for future multi-site trials and dissemination efforts	Tailoring/ tweaking/ refining	*Goal:* Improve adoption *Contextual Reasons:* Peer training and skills & Peer preferences
	Include guidance on documentation	Fillable medical record note templates and an electronic caseload management tool which generates medical record notes based on peers’ in-session notes were developed. Note templates are included in the manual	Tailoring/ tweaking/ refining	*Goal:* Improve feasibility *Contextual Reasons:* VA/clinic regulatory/ compliance
	Develop a caseload management system	A caseload management system including caseload tracking, Veteran reports, a guide to walk through sessions, and session notes was built for the research study with plans to translate it to clinical application	Changes in packaging or materials	*Goal:* Improve feasibility *Contextual Reasons:* Peer perception of intervention
Training and evaluation	Integrate guidelines and tips on how and when to communicate with other staff (e.g., primary care providers)	Concrete guidance was added to the manual about when to communicate with other team members, example language to discuss team communication with Veterans, and tips on how to communicate with other team members	Tailoring / tweaking / refining	*Goal:* Improve fit with VA/clinic setting *Contextual Reasons:* VA/clinic social context
	Clearly define the function and scope of Whole Health STEPS and distinguish it from other existing programs	Explicit discussion of the function and scope of Whole Health STEPS including comparisons to similar existing programs was added to the manual. Marketing materials were also updated accordingly	Tailoring / tweaking / refining	*Goal:* Improve fit with VA/clinic setting *Contextual Reasons:* VA/clinic service structure & VA/Clinic competing demands/ mandates
	Provide concrete guidance on how peers can provide brief, time-limited interventions consistent with primary care models of care	A rationale for brief, time-limited interactions as well as tips on how to keep sessions brief and maintain a strong relationship were added to the manual	Tailoring / tweaking / refining	*Goal:* Improve fit with VA/clinic setting *Contextual Reasons:* VA/clinic service structure
	Streamline processes so it’s simpler and more efficient for both Veterans and peers	In addition to the other adaptations in response to specific suggestions which streamlined Whole Health STEPS (e.g., removing the referral step), the whole manual was simplified to improve clarity	Tailoring/ tweaking/ refining	*Goal:* Improve feasibility *Contextual Reasons:* Peer perception of intervention
	Clarify the types of training that peers need to successfully implement Whole Health STEPS	A list of pre-requisite trainings, recommended trainings, and important local knowledge was added to the manual	Adding elements	*Goal:* Improve fidelity *Contextual Reasons:* Peer training and skills
	Streamline training procedures to minimize staff time	A competency-based training approach was developed such that peers and supervisors can structure training as appropriate as long as peers competently complete role-plays based on a quality checklist. Training materials supporting various learning styles (e.g., vignettes, sample scripts) were developed omitting materials that duplicate existing trainings (e.g., peer training)	Changes in packaging or materials	*Goal:* Improve feasibility & Improve fidelity *Contextual Reasons:* VA/clinic time constraints & Peer training and skills
	Ensure that examples highlight how Whole Health STEPS can support conventional healthcare	Examples were added to the manual to highlight how Whole Health STEPS can support conventional healthcare engagement to address concerns that Whole Health solely focuses on complementary approaches	Tailoring/ tweaking/ refining	*Goal:* Improve fit with VA/ clinic setting *Contextual Reasons:* VA/ clinic social context
	Emphasize that Whole Health STEPS is patient-centered, and patient-driven	Explanations of how Whole Health STEPS supports Veteran identified goals and supports intrinsic motivation through peer support and Whole Health concepts including motivational interviewing consistent skills were added to the manual	Tailoring/ tweaking/ refining	*Goal:* Improve fit with recipients *Contextual Reasons:* Peer perception of intervention & Veteran motivation and readiness
	Ensure there is sufficient rationale for the Whole Health goal assessment so that it is not perceived as “grunt work”	Rationale for peer delivery of the Whole Health goal assessment as part of Whole Health STEPS was added to the manual	Tailoring/ tweaking/ refining	*Goal:* Improve adoption *Contextual Reasons:* Peer perception of intervention
	Provide guidance incorporating other important peer functions and services	Explicit guidance on how to maximize use of the peer skills, including patient navigation, was added. Many peers felt strongly about the importance of peer groups, navigation to peer groups was explicitly included	Tailoring/ tweaking/ refining	*Goal:* Improve adoption *Contextual Reasons:* Peer perception of intervention

When applying the contextual reasons per FRAME, the language has been adapted to enhance clarity (e.g., provider has been changed to peer, recipient has been changed to Veteran, organization/setting has been changed to VA/clinic). The listed classifications for FRAME did not encompass all possible reasons/goals so when necessary, new reasons/goals were generated, these included: Improve adoption, Veteran ease of access/ utilization, Peer training & skills, Improve fit with VA/ clinic setting, and Improve fidelity

Overall, the adaptations made to *Whole Health STEPS* focused on incorporating feedback related to the three cross-cutting themes and addressing the cross-cutting design flaw. Thus, all specific changes were made with the overarching goals to emphasize peer skills, emphasize patient-centered aspects, simplify, and eliminate elements duplicating existing programs. Some changes addressed these overarching goals in multiple ways. For instance, referral was removed as a step in the stepped care process (simplifying the program structure) and made a central element of the design (emphasizing peers’ role as patient navigators). Similarly, Whole Health Coaching (a more complex intervention) was replaced with peer sessions focused on Whole Health which both simplified the program and emphasized peers’ skills. This change allowed us to shorten the recommended length of sessions to give peers more flexibility, and the manual was adapted to include concrete guidance on how to integrate the Whole Health and peer roles. The manual also now includes explicit guidance on the relationship of *Whole Health STEPS* with other VA programs and how peers can incorporate Whole Heath into their work in concordance with the VA’s Whole Health model and peers’ scope of work. Additionally, precise language was used to ensure differentiation of peers as providers from coaches (e.g., all references to “coaching” or “coach” were replaced with more precise and peer-focused language). Another major change that addressed multiple cross-cutting themes was adjusting the step-decision criteria to increase patient-centeredness by increasing Veterans’ role in the decision-making process and adding criteria to step down emphasizing recovery (patient-centeredness and peers’ role) that also streamlined *Whole Health STEPS* delivery by adding the expectation of tapering services and reducing the emphasis on measurement-based decisions.

Additional specific changes focused on other elements including improving marketing and training materials to focus on eventual translation from research trials to clinical roll-out. For instance, peers and supervisors/administrators discussed the importance of having high-quality marketing materials to help with the eventual roll-out, so research trial marketing materials were designed for adaptation for clinical settings and eventual inclusion in an implementation toolkit. Further, specific recommendations for training (e.g., examples and vignettes, experiential training, multi-modal training options) were incorporated into a training plan which will be piloted with training peers for the research trials and adapted for eventual dissemination with an implementation toolkit for training peers in clinical roll-outs.

## Discussion

This rapid qualitative research study focused on understanding future implementation considerations for a novel peer-delivered primary care wellness intervention to inform the intervention design. We found that comprehensive feedback on implementation constructs from CFIR helped us understand the future implementation climate and adapt the *Whole Health STEPS* design to fit within the intended context. We identified cross-cutting themes across implementation constructs and a cross-cutting implementation roadblock; we used these data to directly inform major adaptations to the intervention design. We included a peer as a core member of the research team. We believe that his role administering the interviews may have also enhanced the quality of the data collected. Our findings have implications for researchers, clinicians, and administrators working in this field.

Our project highlights the relevance of implementation constructs in the design phase. Because key implementation constructs relevant to the context and essential elements of the intervention were assessed prior to finalization of the manual, we were able to proactively address stakeholder feedback in a way which would not have been possible to change at a later phase of the study (e.g., changing the number and types of steps involved in the intervention, re-focusing on essential processes) without re-evaluating the efficacy of the revised design. This builds into the existing literature on adaptations and demonstrates how frameworks for adaptations can also be used to support designing for dissemination. Two key processes in designing for dissemination literature which have been identified as gaps are the uptake of frameworks and involving stakeholders in design [30, 47]. Tracking and categorizing adaptations using the FRAME [44] helped us to conceptualize our adaptations in a meaningful way to operationally define design decisions and ensure those decisions aligned with staff feedback. Using the FRAME also helped us understand the potential impact of design decisions by tracking the reasons and goals for each. This may help other researchers focusing on designing for dissemination consider application of adaptations frameworks in addition to other dissemination and implementation frameworks. The addition of an adaptations framework can provide a structure for meaningfully integrating stakeholder feedback into design, and can track design decisions even before a product is fully developed.

Evaluating and understanding the implementation context broadly, including interactions with other programs, meaningfully informed adaptations to *Whole Health STEPS*. Staff feedback pointed out important considerations on multiple levels (e.g., mechanism for peers to track cases, the need to distinguish between key roles), which resulted in adaptations intended to improve acceptability, feasibility, and effectiveness. Feedback also resulted in many adaptations that made Whole Health STEPS clearer and better defined. In fact, these are also some of the aspects highlighted as important considerations in designing for dissemination [30]. We believe the changes will make it better suited for future dissemination and increase the likelihood of success for future implementation efforts. Our results help inform administration and program planning by highlighting some important considerations for new initiatives, including understanding the context for delivery. For organizations undergoing program planning or program development without the resources for an intensive qualitative approach, we suggest incorporating front-line staff and consumer advocates in the design and decision-making process.

Finally, our results identify important and unique considerations for peers’ role in primary care, which can help inform clinical supervision, administrative structure, and the development of other interventions. Peers and supervisors/administrators of peer programs reinforced the importance of emphasizing the peer role and maintaining essential functions of peers (e.g., relationship building, navigation, flexibility) despite the fast-paced setting and stepped-care design. Their feedback builds on prior literature about essential elements of peer services, like trust, social support, and easy accessibility [6] by operationalizing those elements for the primary care setting and this type of intervention in a meaningful way.

### Strengths and limitations

This article has several strengths and limitations which should be acknowledged to put the findings into appropriate context. First, a strength was the national recruitment strategy focused on maximum diversity of VA primary care peer program characteristics likely to influence future implementation of *Whole Health STEPS*, which resulted in a high level of diversity in participants and sites and thus increased likelihood that these results are generalizable at other VA sites. However, given the differences between VA and non-VA settings (including peers’ roles), these results may not generalize to non-VA settings. A strength of the design is the interdisciplinary authorship team and inclusion of a peer as a key member of the research team. However, although peers of our target population are, by definition, Veterans, because of their additional training, recovery status, and status as employees, they differ from non-peer Veteran consumers in some ways that may influence their perspectives on VA interventions. Therefore, a true consumer perspective was not reflected on the research team. The authorship team also reflects multiple VA sites and US regions, which increased understanding of diverse VA settings. However, the authorship team all identify as White, so the perspectives of historically racially and ethnically minoritized communities were not reflected in the scientific planning or analysis level. Individuals identifying as a member of a historically racially and ethnically minoritized community participated in the interviews as research participants. Finally, a strength of the design was the focused questions which allowed us to fully understand relevant concepts with a feasible sample size. However, because these questions were focused exclusively on getting feedback in the context of considering a new primary care peer intervention, participants’ responses should not be interpreted as a reflection of the VA primary care peer program as a whole. Responses were specific to considering the addition of a new program which inherently changes interpretation of the work climate.

## Conclusions

Early feedback from frontline staff, including peer providers, in the design process was crucial to identifying essential modifications that would not have been possible after initial trials without re-evaluating efficacy. Staff feedback, including peer provider feedback, highlighted major themes to guide intervention development and provided specific feedback to address details of the design. As a result, *Whole Health STEPS* was designed to fit within a range of program structures to reduce potential role conflict, emphasize peers’ unique contributions to Veterans’ healthcare, and streamline delivery. Lessons learned can be applied to other peer-delivered interventions for medical settings and other peer-delivered wellness interventions and contribute to the designing for dissemination literature.

### Supplementary Information

**Additional file 1.** 

## Data Availability

The dataset generated and analyzed during the current study are not publicly available due to privacy and confidentiality concerns, but a limited dataset can be made available from the corresponding author on reasonable request.

## Footnotes

1 JH was employed as a Research Coordinator at the time the research was being conducted.
