# Supplementary material for: Integrating stakeholder feedback into the design of a peer-delivered primary care wellness program: A rapid qualitative study
Source: BMC Health Serv Res. 2023 Dec 7;23:1370. doi: 10.1186/s12913-023-10324-x (PMC10701982; doi:10.1186/s12913-023-10324-x)
Supplement: Supplementary file 1 — Additional file 1. [file 12913_2023_10324_MOESM1_ESM.docx]

Online Supplement

This is an online supplement for:

Johnson, E.M., Possemato, K., Chinman, M., True, G., Hedges, J., Hampton, B., Edelman, E.J., Maisto, S.A. *Integrating Stakeholder Feedback Into the Design of a Peer-Delivered Primary Care Wellness Program: A Rapid Qualitative Study.*

Contents

[Stakeholder Interview template 2](#_Toc133494102)

[Informational Handout for Participants 5](#_Toc133494103)

# Stakeholder Interview template

**Instructions**

Bold headers are domain names. Regular text indicates subdomains and descriptions of the goals of each section. Regular text and bold text should not be read aloud. Italicized text indicates what will be said to stakeholders. Although you do not need to read every single italicized prompt to stakeholders, you do need to make sure you have enough information to achieve the goal of each section. If stakeholders start to report on a topic in a different area of the interview (e.g., start talking about intervention characteristics during the implementation climate section) adapt to what they are talking about then go back and ask other prompt questions to get the information for the other sections. If stakeholders have already sufficiently answered a question with a past comment, you can skip those prompts.

**Begin:** *In the first part of the interview, we will ask you questions regarding the primary care peer program at your VA site. Later, we will describe Whole Health STEPS to you in greater detail and ask for your thoughts and opinions on it. I will be asking you about 12 questions overall. We do want to remind you that we value your honest feedback. It is important for us to hear both positive and negative feedback about Whole Health STEPS in order to improve it. We also want to let you know that there may be brief pauses throughout the interview as we take notes.*

| **General Information** | |
| --- | --- |
| *Please tell me about your role as a [peer support provider in primary care/supervisor of a peer support provider in primary care/administrator of a peer support program in primary care].*   - *What is your (your peer’s) role on the primary care team?* - *How long have you been in your role?* | Click or tap here to enter text. |
| **Inner Setting (part 1): Networks & Communications** | |
| *Tell me about your (your peer’s) working relationships with your team members in primary care? With other behavioral health providers and other peers?*   - *What teams do you meet with?* | Click or tap here to enter text. |
| **Implementation Climate (part 1): Tension for Change** | |
| *Has your site implemented Whole Health? Is it meeting the needs of primary care patients and staff?*   1. *How could it be better?* | Click or tap here to enter text. |
| *Are there ways that you think that your peers in primary care program can be better?* | Click or tap here to enter text. |

**Describe Whole Health STEPS:**

*Now let me tell you more about the Whole Health STEPS intervention we want your feedback on. It may be helpful to have your Whole Health STEPS informational handout for this part. Do you have that with you or up on your screen?* *Whole Health STEPS* *is a service under development which is designed for peers in primary care to help Veterans with mental health concerns who are not currently receiving mental health services.* *It is 8-weeks long and includes four levels (or “steps”) of Whole Health services from a Peer.*

**If not already clear:** *Are you familiar with Whole Health?* **If not:** *Whole Health is a VA initiative to improve the health and well-being of Veterans by focusing on whole person care rather than a specific diagnosis and developing personalized care plans based on Veterans’ values. One of the main tools is a Personal Health Inventory (PHI), which is a self-assessment focused on several areas of self-care and professional care to help develop personalized goals.*

*The four steps in Whole Health STEPS are 1) supported self-management (an individual Whole Health STEPS orientation session and use of a self-directed Whole Health tool, like the Personal Health Inventory), 2) brief telephone coaching (weekly 15-minute calls with focused prompts), 3) Whole Health Coaching (weekly hour-long sessions), and 4) referral.* *The role of the Peers is to support Veterans,* *use a tool to find the right “step” for each Veteran, and deliver each Whole Health ”step”.*

*Across all levels, peers will have brief weekly calls* *to check-in and use a tool called the Whole Health Goal Attainment Tool (or WHGAT). The WHGAT helps the peer ask about Veterans’ goals and progress on each of the Whole Health domains* *and uses a rating system to determine whether the Veteran needs extra support and needs to be moved to a higher “step”. An example question is on the back of your handout. The WHGAT only asks about areas within peers’ scope of work including goal setting and goal progress. It is not a clinical or mental health evaluation. Throughout Whole Health STEPS peers will get clinical back-up from other team members according to usual clinic practice for clinical concerns that arise. Do you have any questions about Whole Health STEPS at this point?*

**Questions and general discussion of Whole Health STEPS Prior to eliciting feedback:** Click or tap here to enter text.

| **General Feedback** | |
| --- | --- |
| *Do you have any initial feedback about Whole Health STEPS?*   - *Is there anything that is particularly important for us to include?* - *Are there changes that we should make?* | Click or tap here to enter text. |
| **Intervention Characteristics: Relative Advantage** | |
| *How does Whole Health STEPS compare to other similar existing programs in your setting?* | Click or tap here to enter text. |
| **Outer Setting (part 2): Patient Needs & Resources** | |
| *How well do you think Whole Health STEPS will meet the needs of the Veterans in your clinic?*   1. *In what ways will the intervention meet their needs? E.g. improved access to services? Reduced wait times? Help with self-management? Reduced travel time and expense?* | Click or tap here to enter text. |
| *What barriers will the Veterans in your clinic face to participating in Whole Health STEPS?* | Click or tap here to enter text. |
| **Implementation Climate (part 2): Compatibility** | |
| *How well does the intervention fit with existing work processes and practices in your setting?*   1. *What are likely issues or complications that may arise?* | Click or tap here to enter text. |
| **Characteristics of Individuals: Knowledge & Beliefs about the Intervention** | |
| *How would you feel about doing/supervising Whole Health STEPS?* | Click or tap here to enter text. |
| **Characteristics of Individuals: Self-efficacy** | |
| *What training or materials would you need to do Whole Health STEPS? If you were given that, how confident would you feel doing Whole Health STEPS?* | Click or tap here to enter text. |
| **General Wrap-up** | |
| *Do you have any other final thoughts or opinions about Whole Health STEPS?* | Click or tap here to enter text. |

# Informational Handout for Participants

**Whole Health STEPS**

**(S**tructured **T**iered **E**ngagement with **P**eer **S**upport)

**Whole Health STEPS** is a program that is under development here at the VA. It is designed for peers in primary care to help Veterans with mental health concerns who are not currently receiving mental health services*.* The program is 8-weeks long and includes four levels (or “steps”) of Whole Health services facilitated by a Peer Support Specialist. Peer Support Specialists guide Veterans through the 8-week program and deliver the Whole Health service that each level entails. Their main role is to provide support for Veterans in making progress on wellness goals using the Whole Health model.

Across all levels, Veterans will have brief weekly telephone calls with their peers to evaluate their progress using a **Whole Health Goal Attainment Tool (WHGAT).** The WHGAT is a set of questions that the peer will use to ask about Veterans’ goals and progress on each of the Whole Health domains (see p.2 for an example question). It uses a structured rating system to determine whether the Veteran needs extra support and needs to be moved to a higher “step.” Veterans who are not making progress at any of the given levels will be stepped-up to a higher level of care. The level of support provided will be determined based on Veterans’ progress and will be stepped-up until Veterans receive the level of support they need.


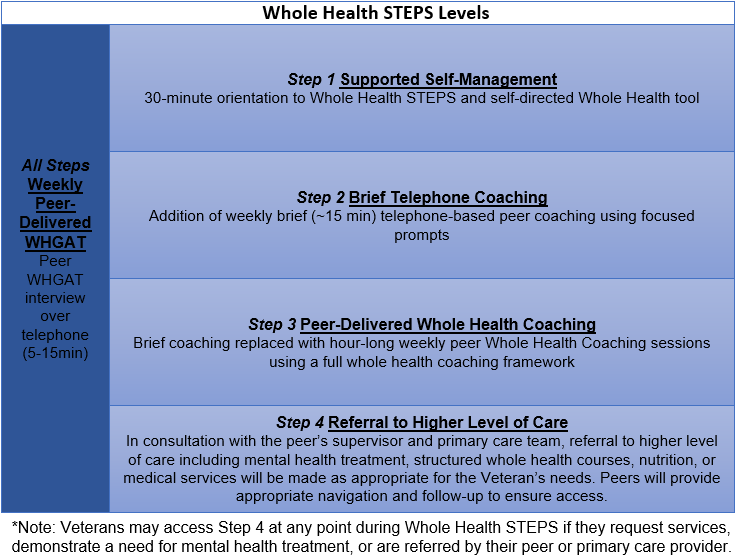


**Example WHGAT Question:**

1. Food and Drink (or nourish and fuel) is eating healthy, balanced meals with plenty of fruits and vegetables each day, drinking enough water, and limiting sodas, sweetened drinks, and alcohol. Are you thinking about or working on making improvements to this area?
   - *No, GAS = 0,* Move to next domain.
   - *Yes,* Do you have a specific goal?
     - *Yes.*
2. What is your goal? ________________________
3. What have you done to work on your goal in the last week? __________________________________________________ *Document and ask follow-up questions as necessary to rate on 1-10 scale below:*
4. *Has a goal but have not done anything to work on it.*
5. *Has talked with a support person (family, friend, doctor, case manager, etc.) about goal*
6. *Has researched ways of achieving goal without help from others*
7. *Has made specific plans to reach goal but have not taken any steps to work on goal*
8. *Has taken at least one step towards achieving goal and made some progress or preparation for working toward goal (1-25% of the way toward goal)*
9. *Is working towards goal and made good progress (26-50% of the way toward goal)*
10. *Is working towards goal and made very good progress*

*(51-75% of the way toward goal)*

1. *Has taken a lot of steps towards goal and almost achieved it (76-99% of the way toward goal)*
2. *Has achieved goal (100%)*
3. *Has exceeded initial goal*
   - - *No, GAS = 0, move to next domain.*

*Note: The WHGAT was designed to only ask about areas within peers’ scope of work including goal setting and goal progress and is NOT a clinical or mental health evaluation. Peers will get clinical back-up from other team members according to usual clinic practice for any mental health or clinical concerns that arise in Whole Health STEPS.
